# Tumor burden affects the progression pattern on the prognosis in patients treated with sorafenib

**DOI:** 10.3389/fonc.2024.1405178

**Published:** 2024-04-23

**Authors:** Jun Sun, Dongdong Xia, Wei Bai, Xiaomei Li, Enxing Wang, ZhanXin Yin, Guohong Han

**Affiliations:** ^1^ Department of Liver Disease and Digestive Interventional Radiology, National Clinical Research Center for Digestive Diseases and Xijing Hospital of Digestive Diseases, Air Force Medical University, Xi’an, Shaanxi, China; ^2^ Department of Liver Diseases and Interventional Radiology, Digestive Diseases Hospital, Xi’an International Medical Center Hospital, Northwestern University, Xi’an, Shaanxi, China

**Keywords:** HCC, sorafenib, progression pattern, tumor burden, interaction

## Abstract

The progression pattern of tumors has an impact on the survival of patients with advanced hepatocellular carcinoma (HCC) and has been applied in the design of clinical trials for multiple second-line drugs. Previous research results have been contradictory, and the clinical impact of different progression patterns and their role in survival are still in question.

**Purpose:**

The study aims to analyze the impact of different progression patterns and tumor burden size on survival of HCC patients, as well as their interactions, through a retrospective cohort study.

**Patients and methods:**

The study involved 538 patients who had undergone treatment with sorafenib and had shown radiographic progression. The progression pattern was analyzed using Cox regression by including an interaction term between progression pattern and tumor burden, which was then visualized through a graphical analysis. Tumor burden was categorized into low, medium, and high subgroups based on the six-and-twelve criteria, allowing for an exploration of the effect of progression pattern on survival in different tumor burden situations.

**Results:**

Compared to patients with only intrahepatic progression (NIH/IHG) with an overall survival (OS) of 14.1/19.9 months and post-progression survival (PPS) of 8.1/13.1 months respectively, patients with extrahepatic lesions (NEH/EHG) had worse overall and postprogressive survival (OS: 9.3/9.2 months, PPS: 4.9/5.1 months). The hazard ratio for extrahepatic progression (NEH/EHG) compared to intrahepatic progression (NIH/IHG) at low, medium, and high tumor burden were [HR 2.729, 95%CI 1.189-6.263], [HR 1.755, 95%CI 1.269-2.427], and [HR 1.117, 95%CI 0.832-1.499], respectively.

**Conclusion:**

The study concluded that the interaction between the tumor progression patterns and tumor burden significantly affects the prognosis of HCC patients. As the tumor burden increases, the sensitivity of the patient’s risk of death to the progression pattern decreases. These findings are valuable in personalized treatment and trial design.

## 1 Introduction

Tumor progression is generally considered a discouraging event and is seen as a reflection of treatment failure that requires shifting to another treatment approach (1–3). However, in fact, progression may have different patterns, and the progression pattern is an important factor that affects the subsequent survival of liver cancer patients, distinguishing them according to the location of lesion progression (4–9). The 2020 trial design and endpoints in hepatocellular carcinoma: AASLD (American Association for the Study of Liver Diseases) consensus conference mentioned that the trial design of second-line treatment for advanced hepatocellular carcinoma should take into account the different progression patterns after first-line treatment and this has been applied to the clinical trial design of multiple second-line drugs (4, 7–10). Stratifying patients based on their progression patterns provides a validated predictor of treatment outcomes and has become a relevant parameter for informing patients, designing and analyzing clinical trials. In clinical trials, reasonable survival assumptions are key to determining the potential impact of new drugs on expected lifespan.

However, past related research has yielded conflicting results (**Table 1**) (11–17). In 2013, Maria Reig et al. for the first time explored the relationship between survival and progression patterns in patients who progressed after receiving sorafenib treatment and had good liver function and performance scores, and found that new extrahepatic lesions (NEH) were a poor prognostic factor, providing evidence that different types of progression should be considered when stratifying patients and emphasized the need for further analysis and clarification of the prognostic significance of different progression types (11). In 2015, Massimo Colombo et al. found that although NEH was an independent prognostic factor, the post-progression survival of patients with extrahepatic growth (EHG) was similar to that of NEH patients, with respective time periods of 3.2 and 3.1 months (12). In the sub-analysis of the SORAMIC trial in 2020, NEH was not a poor prognostic factor (with respective median survival times of 14.8 and 14.9 months compared to overall survival), and only lung metastasis was a poor prognostic factor (7.6 months) (15). In SIRT treatment, Bruno Sangro et al. found that the NEH or NIH progression patterns represented a poor prognosis (16). Of course, more research points to NEH as an independent prognostic factor.

**Table 1:** Previous studies on the prognostic significance of progression patterns in patients with advanced hepatocellular carcinoma.

Author	Year	Treatment	Independent prognostic factor
Maria Reig	2013	sorafenib	NEH
Massimo Iavarone	2015	sorafenib	NEH
Yi-Hsiang Huang	2015	sorafenib	NEH
Sadahisa Ogasawara	2016	sorafenib	NEH
Kerstin Schütte	2020	sorafenib/sorafenib plus SIRT	New pulmonary metastases
Bruno Sangro	2020	SIRT	NEH/NIH
Maria Reig	2020	ramucirumab	NEH

SIRT, selective internal radiation treatment; NEH, new extrahepatic lesion and/or vascular invasion; NIH: new intrahepatic lesion.

Advanced hepatocellular carcinoma is characterized by significant heterogeneity, with tumor burden, metastases, liver function reserve, and overall health status all significantly impacting patient survival time and quality of life (18). Moreover, studies have shown that even in patients with liver cancer accompanied by extrahepatic metastases, more than 80% of deaths are attributed to intrahepatic tumor progression, with liver failure resulting from late-stage progression being the main cause of death (19). Previous studies have also found that as tumor burden increases, the correlation between imaging response and survival rate after Transarterial chemoembolization (TACE) treatment weakens, possibly due to a balance between the positive impact of imaging response and the negative impact on liver function in patients with high tumor burden (20). Therefore, in the progression patterns of advanced liver cancer, can intrahepatic tumor burden affect the prognosis of progression patterns, with the negative impact on liver function resulting from the rapid deterioration in patients with high tumor burden being consistent with the negative impact of extrahepatic lesion progression?

Therefore, we propose the following hypothesis: In cases of low tumor burden, the appearance of extrahepatic lesion progression (NEH/EHG) signifies poor prognosis, but with increasing tumor burden, the sensitivity of extrahepatic lesion progression prognosis gradually decreases.

## 2 Patients and methods

### 2.1 Study population

This study retrospectively included 1048 patients with HCC who received sorafenib at our Center from January 2010 to October 2019, including patients with advanced HCC or those who were resistant to TACE therapy. Diagnosis was made by imaging or histological assessment according to American Association for the Study of Liver Diseases (AASLD) or European Association for the Study of Liver Diseases (EASL) guidelines. Exclusion criteria included: (1) accompanied by other malignant tumors; (2) Received any local treatment (ablation or TACE, etc.) within 4 weeks prior to the initiation of sorafenib; (3) Child-Pugh grade C patients; (4) Patients with ECOG physical status score over 2 points; (5) Patients who lacked progressive imaging until the last follow-up.

Patients received an initial dose of sorafenib of 400mg BID and the dose was adjusted in the event of intolerable adverse reactions. In the event of intolerable toxicity, the dose of sorafenib is reduced accordingly, or even temporary or permanent discontinuation of sorafenib therapy, but patients are usually encouraged to continue sorafenib therapy when adverse reactions can be tolerated.

### 2.2 Data collection

Commonly variables collected for the analysis were baseline demographic patient characteristics, radiological images and serum parameters.

Multiphase computed tomography (CT) or dynamic enhanced magnetic resonance imaging (MRI) imaging was performed before treatment initiation and every 8 weeks after treatment, and tumor response (complete response, partial response, disease stabilization, tumor progression) was evaluated according to modified response evaluation criteria in solid tumors (mRECIST) (21).

The progress time of imaging evaluation of patients was recorded, and the type of progress was registered: IHG: ≥20% increase in the size of intrahepatic lesions compared with baseline (intrahepatic growth); NIH: new intrahepatic lesion; EHG: ≥20% increase in the size of extrahepatic lesions compared with baseline (extrahepatic growth); NEH: new extrahepatic lesion and/or vascular invasion (11).

Tumor burden was categorized into low, medium, and high subgroups based on the six-and-twelve criteria (The sum of tumor numbers and maximum diameters was delimited by truncation values of six and twelve) (22).

The primary outcome points were overall survival and post-progression survival. Follow-up was conducted by a professional clinical follow-up team every 8 weeks until death or the last follow-up date or contact was lost. On October 9, 2021, a final follow-up was conducted and a final survival assessment was made for all patients. The procedures followed in this study conformed to the ethical guidelines of the Helsinki Declaration of 1975 and were approved by the Ethics Committee of Xijing Hospital (Xi’an, China). According to institutional guidelines, all patients signed a written informed consent for treatment and to provide their clinical data in subsequent research before receiving sorafenib therapy.

### 2.3 Statistical analysis

Quantitative variables are presented as means with standard deviation (SD) or medians with interquartile range median with interquartile range, and were compared by Student’s t test or Mann-Whitney U test. Categorical variables were presented as absolute and relative frequencies and compared by Chi-square test or Fisher’s exact test. The interaction multiplicative terms of progression pattern and tumor load were included in COX regression, and the interaction was analyzed by drawing viewable views. Survival curves were plotted using the Kaplan-Meier (KM) method. For all analyses, a corresponding p value less than 0.05 was considered statistically significant. All calculations were performed with SPSS v22 (SPSS Inc., Chicago, IL) and R version 4.1.0 (R Foundation for Statistical Computing, Vienna, Austria).

## 3 Results

### 3.1 Baseline characteristics

**Figure 1:**
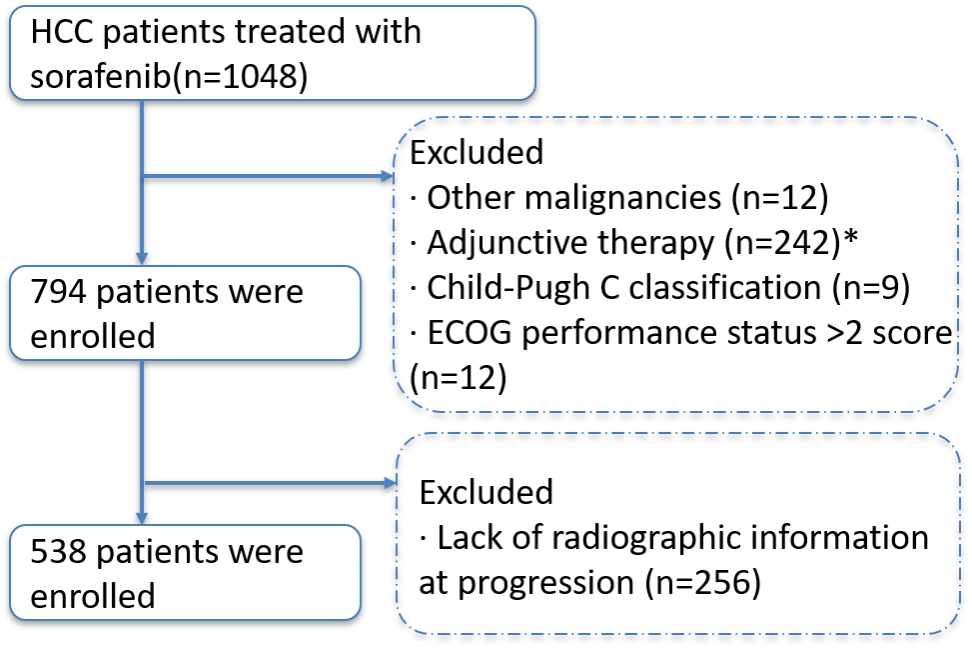
Selection flow diagram. **Comment:** * Received any local treatment (ablation or TACE, etc.) within 4 weeks prior to the initiation of sorafenib.

A total of 538 patients with hepatocellular carcinoma that progressed after receiving sorafenib were included in this study (**Figure 1**). Chronic hepatitis B virus infection was the main cause in 463 patients (86.1%). The patients were mainly with medium-high tumor burden: 52 patients (9.7%) in low-load group, 251 patients (46.7%) in medium-load group, and 235 patients (43.7%) in high-load group. In the mode of progression, there were 246 cases (45.7%) of intrahepatic lesions, 148 cases (27.5%) of new extrahepatic lesions, 103 cases (19.1%) of new intrahepatic lesions, and 41 cases (7.6%) of extrahepatic lesions. The baseline data of all patients are shown in **Table 2**.

**Table 2:** Demographic and baseline characteristics of the patients.

Variables	Total cohorts(n=538)
Age, y (SD)	52.96(11.12)
Males (%)	457(84.9%)
Etiology (%)	
HBV	463(86.1%)
HCV	18(3.3%)
others	57(10.6%)
BCLC-C (%)	422(78.4%)
Largest tumour size, mm (IQR)	8.4(6.0-11.7)
Tumour number (IQR)	2(1-3)
Six-and-twelve (%)	
Low	52(9.7%)
Intermediate	251(46.7%)
High	235(43.7%)
Progression pattern (%)	
NIH	103(19.1%)
IHG	246(45.7%)
EHG	41(7.6%)
NEH	148(27.5%)
ECOG PS (%)	
0	258(48.0%)
1	261(48.5%)
2	19(3.5%)
ALBI 1 (%)	250(46.5%)
Child-Pugh A (%)	415(77.1%)
AFP, ng/ml (IQR)	449.3(20.44-11693)
NLR (IQR)	2.82(1.89-4.20)
ALB, g/L (SD)	39.37(5.04)
Bilirubin (μmol/L) (%)	16.80(12.50-22.50)
AST, U/L (IQR)	49(33-79)
INR (IQR)	1.09(1.02-1.17)
Creatinine, µmol/l (IQR)	86(76-96)
Macrovascular invasion (%)	230(42.8%)
Extrahepatic spread (%)	231(42.9%)
Ascites (%)	101(18.8%)

AFP, alpha-fetoprotein; ALB, albumin; ABLI, albumin-bilirubin; AST, aspartate aminotransferase; BCLC, Barcelona Clinic Liver Cancer; HBV, hepatitis B virus; INR, international normalized ratio; IQR, interquartile range; PS, performance status.

### 3.2 Survival analysis

**Figure 2:**
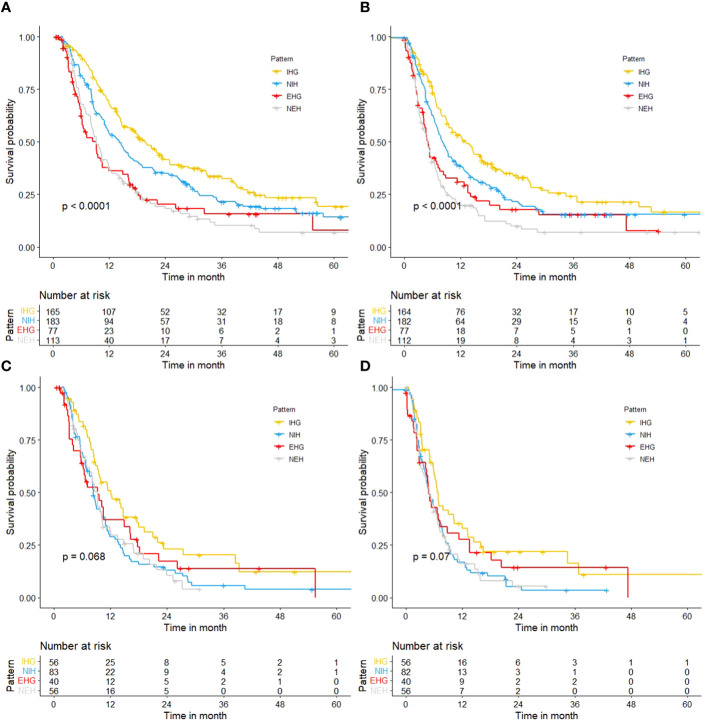
Kaplan-Meier curves. **(A)** OS of different progression patterns in total cohort; **(B)** PPS of different progression patterns in total cohort; **(C)** OS of different progression patterns in patients with a high tumor burden; **(D)** PPS of different progression patterns in patients with a high tumor burden.

The median follow-up was 11.8 months (IQR 6.2–24.7 months). In the general population, the median survival of patients with different progression modes (NEH, EHG, NIH, IHG) was 9.3 (95%CI 8.1-11.6) months, 9.2 (95%CI 6.2-11.8) months, 14.1 (95%CI 11.3-16.6) months, and 19.6 (95%CI 15.8-24.8) months, respectively (*p*<0.001); The post-progression survival was 4.9 (95%CI 3.8-6.4) months, 5.1 (95%CI 4.2-7.5) months, 8.1 (95%CI 7.0-10.1) months, 13.1 (95%CI 9.5-16.5) months, respectively (*p*<0.001) (**Figure 2**).

But in people with a high tumor burden, the median survival of patients with different progression modes (NEH, EHG, NIH, IHG) was 9.2 (95%CI 7.0-10.4) months, 9.3 (95%CI 6.2-16.3) months, 8.3 (95%CI 7.5-10.8) months, and 12.1 (95%CI 9.3-17.9) months, respectively (*p=*0.068); The post-progression survival was 4.9 (95%CI 3.2-7.0) months, 4.8 (95%CI 4.2-11.3) months, 5.0 (95%CI 4.1-6.9) months, 6.6 (95%CI 5.9-12.0) months (*p=*0.07). and it was difficult to distinguish survival based on the mode of progression. The effect of progression patterns on patient survival was no longer statistically significant.

### 3.3 Interaction analysis

**Figure 3:**
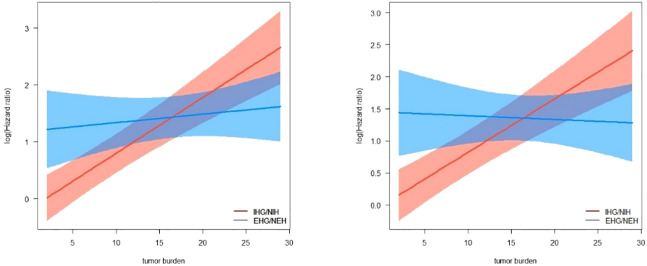
Interactive visualization of the interaction between tumor burden and progression patterns with OS/PPS as the endpoint.

The multivariate Cox regression analysis included factors related to the guidelines recommended grouping criteria for clinical trials, in addition to tumor load and the multiplicative interaction terms of tumor load and pattern of progression (progression limited to intrahepatic/extrahepatic progression). The results showed that MVI, AFP, tumor burden, progression pattern and the multiplicative interaction terms of tumor load and mode of progression were statistically significant for the prognosis of patients (*p*< 0.001) (**Table 3**). A restricted cube plot (**Figure 3**) shows that the risk of death in patients with liver-limited progression (NIH/IHG) increases with the enlarging in tumor load until the risk is close to that in patients with extrahepatic progression at high tumor load.

**Table 3:** Analysis of interaction between tumor burden and progression pattern.

Variables	HR (95%CI)	*p* value
	OS	
ECOG PS (≥1 vs 0)	1.134(0.922-1.393)	0.233
MVI (yes vs no)	1.472(1.184-1.830)	<0.001
AFP (>400ng/ml vs ≤400ng/ml)	1.574(1.285-1.928)	<0.001
Tumor burden	1.103(1.072-1.928)	<0.001
Progression pattern (Intrahepatic vs extrahepatic)	3.918(2.177-7.050)	<0.001
Interaction between tumor burden and progression pattern	0.920(0.878-0.965)	<0.001
	PPS	
ECOG PS (≥1 vs 0)	1.060(0.863-1.302)	0.579
MVI (yes vs no)	1.299(1.044-1.617)	0.019
AFP (>400ng/ml vs ≤400ng/ml)	1.648(1.343-2.022)	<0.001
Tumor burden	1.087(1.058-1.117)	<0.001
Progression pattern (Intrahepatic vs extrahepatic)	4.311(2.408-7.720)	<0.001
Interaction between tumor burden and progression pattern	0.915(0.873-0.958)	<0.001

AFP, alpha-fetoprotein; MVI, Macrovascular invasion.

### 3.4 Relationship between progression patterns and mortality risk under different tumor burden

Multivariate COX regression analysis was performed according to the clinical trial grouping criteria recommended by the guidelines. The pattern of progression was found to be independent prognostic factor *p*<0.001 for both overall median survival and post-progression survival (**Table 4**). Patients with extrahepatic progression or new extrahepatic progression had a 1.549 times higher risk of death (95%CI 1.256-1.909).

**Table 4:** Multivariate cox regression analysis under different tumor loads.

Variables	OS		PPS	
	HR (95%CI)	*p* value	HR (95%CI)	*p* value
Total				
ECOG (≥1 vs 0)	1.283(1.047-1.571)	0.160	1.177(0.960-1.443)	0.117
MVI (yes vs no)	1.629(1.313-2.020)	0.001	1.435(1.155-1.782)	0.001
AFP (>400ng/ml vs ≤400ng/ml)	1.702(0.391-2.084)	<0.001	1.728(1.410-2.117)	<0.001
Progression pattern (Intrahepatic vs extrahepatic)	1.549(1.256-1.909)	<0.001	1.558(1.261-1.925)	<0.001
Low				
ECOG (≥1 vs 0)	3.243(1.466-7.174)	0.004	4.008(1.676-9.586)	0.002
MVI (yes vs no)	0.667(0.191-2.330)	0.526	0.495(0.141-1.739)	0.273
AFP (>400ng/ml vs ≤400ng/ml)	3.706(1.707-8.046)	0.001	3.853(1.693-8.768)	0.001
Progression pattern (Intrahepatic vs extrahepatic)	2.729(1.189-6.263)	0.018	1.776(0.736-4.282)	0.201
Intermediate				
ECOG (≥1 vs 0)	1.151(0.844-1.571)	0.375	1.069(0.782-1.460)	0.676
MVI (yes vs no)	1.621(1.148-2.289)	0.006	1.439(1.014-2.043)	0.042
AFP (>400ng/ml vs ≤400ng/ml)	1.680(1.234-2.287)	0.001	1.744(0.281-2.374)	<0.001
Progression pattern (Intrahepatic vs extrahepatic)	1.755(1.269-2.427)	0.001	1.913(1.378-2.654)	<0.001
High				
ECOG (≥1 vs 0)	1.059(0.787-1.424)	0.706	0.992(0.737-1.336)	0.959
MVI (yes vs no)	1.351(1.000-1.824)	0.050	1.185(0.878-1.598)	0.268
AFP (>400ng/ml vs ≤400ng/ml)	1.455(1.088-1.947)	0.012	1.469(1.096-1.968)	0.010
Progression pattern (Intrahepatic vs extrahepatic)	1.117(0.832-1.499)	0.462	1.117(0.832-1.498)	0.462

AFP, alpha-fetoprotein; MVI, Macrovascular invasion.

However, when subgroups were differentiated according to tumor load, it was found that the sensitivity of patients’ risk of death to the pattern of progression decreased with increasing tumor load. The hazard ratio for extrahepatic progression (NEH/EHG) compared to intrahepatic progression (NIH/IHG) at low, medium, and high tumor burden were [HR 2.729, 95%CI 1.189-6.263], [HR 1.755, 95%CI 1.269-2.427], and [HR 1.117, 95%CI 0.832-1.499], respectively.

## 4 Discussion

Based on a retrospective analysis of 538 patients with hepatocellular carcinoma who progressed after treatment with sorafenib, we found that there was a significant interaction between tumor burden and progression pattern on the outcome of hepatocellular carcinoma. At the same time, the changes of the relationship between death risk and progression pattern of patients under different tumor burden were further analyzed using tumor burden as stratification condition. Our study partly explains why previous studies have reached different conclusions about the progression pattern of advanced liver cancer tumors, in which tumor burden was not included in the analysis (11–17).

According to the tumor burden model established previously by our research group (six-and-twelve) (22), we divided the tumor burden into three subgroups of low, medium and high, and found that as the tumor burden increases, the sensitivity of the patient’s risk of death to the progression pattern decreases: In patients with low and moderate tumor loads, patients with extrahepatic progression (NEH/EHG) had a significantly worse prognosis than those with intrahepatic progression (NIH/IHG). In patients with high tumor burden, the progression pattern no longer significantly stratified patient survival. Meanwhile, in patients whose progression was limited to the liver (NIH/IHG), median survival of different subgroups of at-risk individuals was significantly differentiated based on tumor burden; However, it is difficult to distinguish different risk groups based on tumor burden when there is extrahepatic lesion progression. This may be because the negative effects of rapid deterioration of liver function in patients with high tumor burden after progression are similar to the negative effects of extra-hepatic lesion progression.

With the development of first-line therapy such as molecular targeted therapy and immunotherapy, the therapeutic strategy of second-line therapy is also about to change dramatically (23, 24). Appropriate stratification factors should be considered in the trial design of second-line therapy for advanced liver cancer, otherwise patient characteristics may influence clinical trial results in patients with treatment failure (4). Our study suggests that the interaction between progression patterns and tumor burden in trial design should be fully considered in trial design and personalized treatment.

In our study, high tumor burden and medium tumor burden together accounted for more than 90% of the total population, which is consistent with the popular situation of our country, and the influence of tumor burden on our patients is more important (25). In the general population, overall survival and post-progression of new and extra-hepatic lesions were poor (OS: 9.3, 9.2; PPS: 4.9 months, 5.1 months). For patients with high tumor burden, regardless of intrahepatic progression or extrahepatic progression, the survival time after progression was only about half a year (4.9 months, 4.8 months, 5.0 months, 6.6 months). Therefore, for patients with higher tumor burden and better liver function reserve, active treatment with higher objective remission rate and disease control rate is recommended, because the objective remission rate of sorafenib is low, once the tumor progression is not controlled, it is difficult to bring better survival benefits to patients. At the same time, our study found that AFP had significant prognostic significance in different subgroups, and its prognostic value and significance should be further explored.

Advanced liver cancer has considerable heterogeneity, and tumor burden, metastasis, liver function reserve, and systemic condition all have considerable influence on the survival time and quality of life of patients (18, 26). However, the degree of influence of these factors varies. In 2018, Giannini et al. used clinical characteristics to classify heterogeneous BCLC stage C patients. In a retrospective analysis of 835 patients with stage C BCLC, median overall survival was significantly different based on criteria leading to advanced tumor stage (ECOG score 1-2, macrovascular infiltration, or extrahepatic spread) (18). At the same time, these factors will also influence each other and interact with each other, instead of simply adding or subtracting. However, the current research mainly divides patients into different groups according to a certain factor. In the follow-up studies of prognostic factors, faced with complex individuals, we should study more the interaction between multiple factors, rather than just explore the independent prognostic effect of a single factor.

Intrahepatic tumor burden, macrovascular invasion and extrahepatic metastasis, as well as severe liver function impairment, are key factors for poor prognosis, and these factors are often reflected in multiple clinical prediction models (27–32). However, tumor burden is rarely considered in the trial design of second-line therapy, possibly because its role is often overwritten by adverse prognostic factors such as liver function and extrahepatic metastasis (7–10, 33, 34). Patients with high tumor burden are more likely to suffer from rapid deterioration of liver function or even liver failure, resulting in death, after the progression of intrahepatic tumor, and this risk should not be ignored (19). The liver function and tumor burden can be in a certain interaction between need further exploration in the future. On the one hand, normal liver tissue is affected in patients with large tumor burden, and people with poor liver function should be more than patients with small tumor burden. On the other hand, as the patient’s liver function declines, the sensitivity of tumor burden to survival prognosis may also gradually decrease.

The study also has some limitations. First, the risk of selection bias is inevitable in observational studies. Secondly, the cause of Chinese patients is mainly hepatitis B, and the tumor burden is also relatively high, which has some differences with other areas of tumors, and needs to be further verified in multi-center and other areas. At the same time, we only considered the interaction between tumor burden and progressive mode, and did not consider the interaction between tumor burden and progressive mode and liver function. This part of work needs to be further explored in the future.

## 5 Conclusion

In conclusion, interaction between the tumor progression patterns and tumor burden significantly affects the prognosis of HCC patients. As the tumor burden increases, the sensitivity of the patient’s risk of death to the progression pattern decreases. Therefore, the interaction between progression mode and tumor burden should be fully considered in trial design and personalized treatment.

## Data availability statement

The data that support the findings of this study are available on request from the corresponding author, Guohong Han, upon reasonable request. Requests to access these datasets should be directed to Jun Sun, 438831443@qq.com.

## Ethics statement

The studies involving humans were approved by Ethics Committee of Xijing Hospital. The studies were conducted in accordance with the local legislation and institutional requirements. Written informed consent for participation was not required from the participants or the participants’ legal guardians/next of kin in accordance with the national legislation and institutional requirements.

## Author contributions

JS: Writing – review & editing, Writing – original draft, Visualization, Software, Methodology, Investigation, Data curation, Conceptualization. DX: Writing – review & editing, Validation, Supervision, Software, Project administration, Methodology, Investigation, Conceptualization. WB: Writing – review & editing, Supervision, Resources, Project administration, Methodology, Investigation, Formal Analysis, Data curation, Conceptualization. XL: Writing – review & editing, Resources, Investigation, Data curation. EW: Writing – review & editing, Methodology, Investigation, Data curation, Conceptualization. ZY: Writing – review & editing, Resources, Project administration, Methodology, Investigation, Data curation, Conceptualization. GH: Writing – review & editing, Validation, Supervision, Software, Resources, Project administration, Methodology, Investigation, Funding acquisition, Formal Analysis, Data curation, Conceptualization.
